# Laparoscopic Resection of Para-Aortic Mass at the Aortic Bifurcation: An Atypical Presentation of a Tailgut Cyst

**DOI:** 10.7759/cureus.62391

**Published:** 2024-06-14

**Authors:** Andy Wang, Jiddu A Guart, Danielle Li, Trenton Taros, Hongyi Cui

**Affiliations:** 1 Surgery, University of Massachusetts Chan Medical School, Worcester, USA; 2 General Surgery, University of Massachusetts Chan Medical School, Worcester, USA

**Keywords:** aortic bifurcation, laparoscopic resection of para-aortic mass, para-aortic mass, laparoscopic resection, tailgut cysts, para-aortic

## Abstract

We review the case of a 43-year-old white male who presented with an enlarging pulsatile mass in the periumbilical region. Diagnostic imaging revealed an 8-cm heterogeneous mass abutting the left iliac artery at the aortic bifurcation. Due to the patient’s persistently elevated blood pressure and elevated serum and urine catecholamines, a neuroendocrine tumor was suspected. Laparoscopic resection was performed and was well tolerated. However, the mass was characterized as a tailgut cyst upon pathological examination. This case highlights the utility of laparoscopy for the removal of large para-aortic masses, which can be achieved in a safe fashion by an experienced surgeon. In addition, this case highlights the importance of differential diagnoses in surgeries due to the occurrence of unexpected outcomes.

## Introduction

Para-aortic masses present a unique challenge in surgical management due to their proximity to major vascular structures, making precise and minimally invasive approaches particularly valuable. The emergence of laparoscopic techniques has revolutionized the surgical treatment of these masses, offering reduced morbidity and faster recovery times compared to traditional open surgery. This case report discusses the laparoscopic resection of a para-aortic mass located at the aortic bifurcation, identified as an atypical presentation of a tailgut cyst.

Tailgut cysts, or retrorectal cystic hamartomas, are rare congenital lesions that arise from remnants of the embryonic tailgut [1]. These cysts are typically located in the presacral region, but their occurrence near the aortic bifurcation is uncommon. We discuss diagnostic challenges regarding tailgut cysts in atypical locations. While the initial workup suggested a neuroendocrine tumor based on imaging and lab findings, analysis of the specimen ultimately confirmed the mass to be a tailgut cyst. The current literature on tailgut cysts primarily highlights their diagnosis and management in the more common presacral location, with limited reports detailing the various surgical techniques in the resection of a tailgut cyst in the para-aortic region [2,3].

The advent of laparoscopic surgery has significantly impacted the approach to para-aortic masses. Traditional open surgery, while effective, is associated with greater postoperative pain, longer hospital stays, and increased recovery time. In contrast, laparoscopic techniques offer enhanced visualization of the surgical field, reduced postoperative discomfort, and quicker patient recovery. Studies comparing open versus laparoscopic resection of para-aortic masses indicate that laparoscopic methods are associated with shorter operative times, less intraoperative blood loss, and fewer complications [4].

This case report adds to the limited body of literature on the laparoscopic management of para-aortic tailgut cysts, demonstrating the utility of using a minimally invasive approach in such atypical cases. By detailing this case, we aim to provide insights into the diagnosis and surgical management of rare para-aortic presentations of tailgut cysts, contributing to the evolving landscape of minimally invasive surgery in complex anatomical regions.

## Case presentation

**Figure 1:**
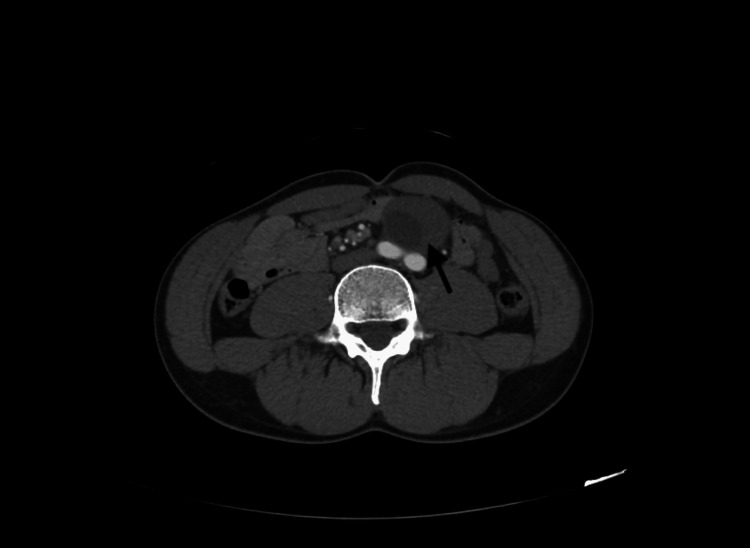
CT scan: axial view of para-aortic mass measuring 6.4 × 8.3 × 8.8 adjacent to the aortic bifurcation

**Figure 2:**
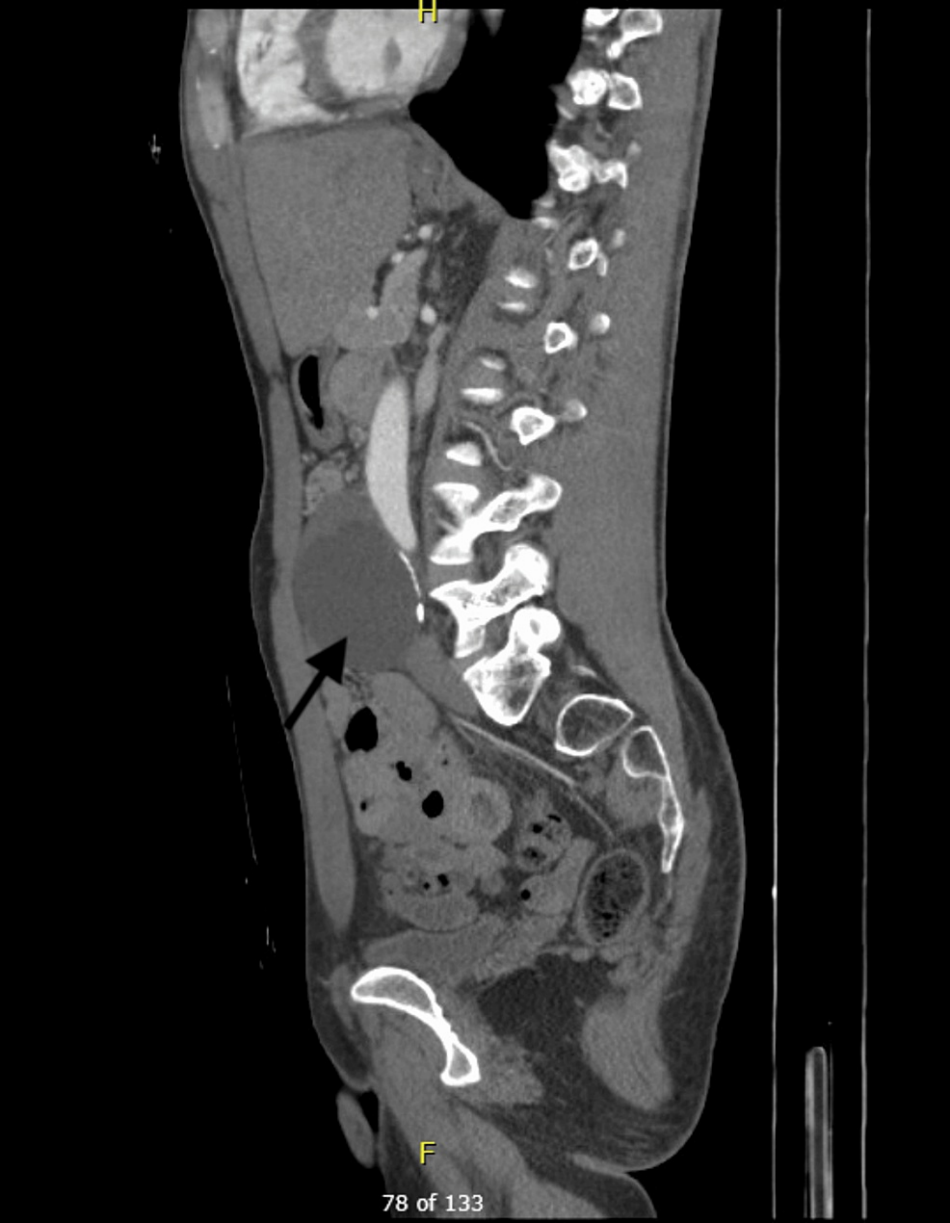
CT scan: sagittal view of para-aortic mass with posterior calcifications

**Figure 3:**
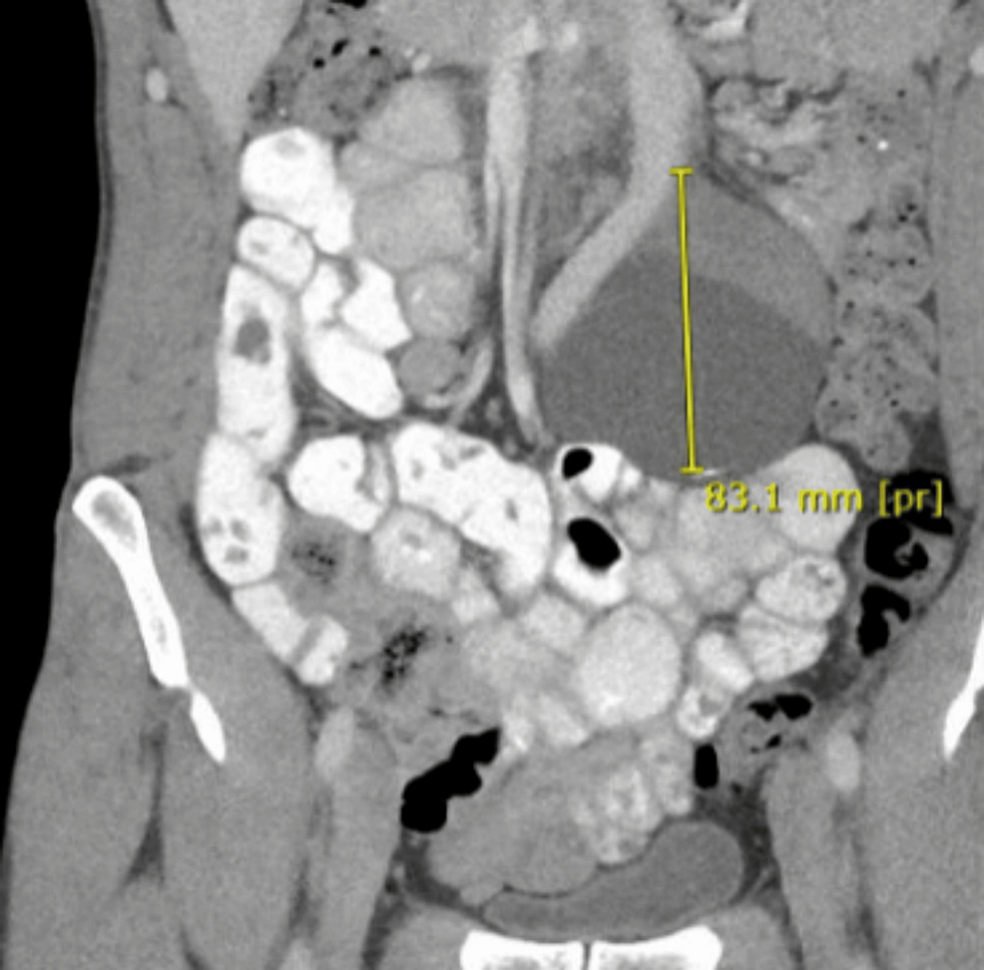
CT scan: coronal view of the para-aortic mass demonstrating its proximity to the aortic bifurcation and its heterogeneous components

**Figure 4:**
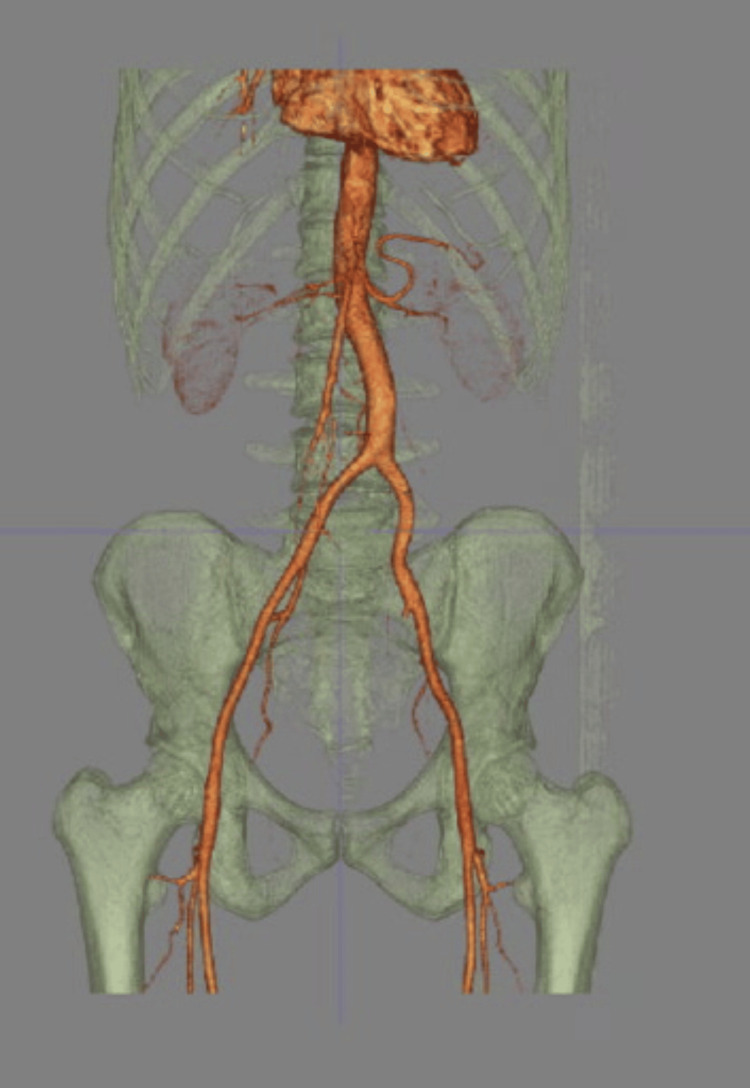
Arterial reconstruction demonstrating lack of communication of the para-aortic mass with the aorta

A 43-year-old, healthy white male presented to the general surgery clinic complaining of vague abdominal discomfort for two years. Two years prior, a CT scan revealed an 8.1-cm hypodense lesion with irregular calcifications abutting the aortic bifurcation and bilateral common iliac arteries (Figures 1-3). Due to concerns about aortic aneurysms, the patient was referred to vascular surgery. Further evaluation with a CT angiogram and vascular duplex showed no flow into the mass (Figure 4). At a later date, the mass was aspirated by interventional radiology, yielding 115 mL of clear fluid, which was nondiagnostic. Subsequently, the patient was referred back to general surgery.

At our clinic, the patient reported gradual enlargement of the mass. He mentioned no symptoms of dizziness, palpitations, headaches, or syncope and denied weight loss, fatigue, blurry vision, cough, or chest pain. However, he was found to have new-onset mild hypertension. During the physical exam, a pulsatile mass was felt in the abdomen. A possible neuroendocrine etiology was suspected, and serum and urine catecholamine levels were drawn. Both free metanephrine and normetanephrine were elevated, along with plasma norepinephrine and total catecholamine levels. While urine metanephrine levels were mildly elevated, urine epinephrine, norepinephrine, and normetanephrine were within normal limits. At this point, there was a high suspicion of a neuroendocrine tumor, such as a paraganglioma of the organ of Zuckerkandl, due to its location and lab results. It was decided to proceed with laparoscopic resection of the mass.

**Figure 5:**
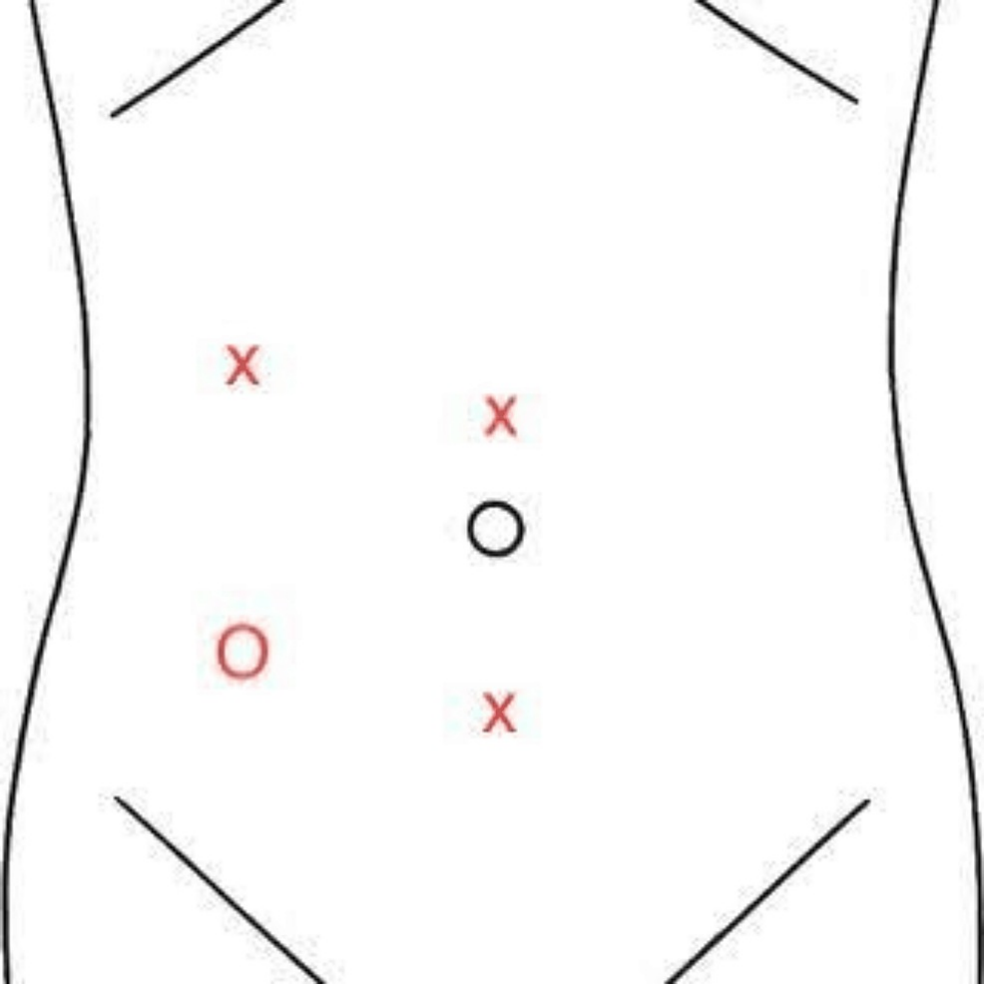
Placement of trocars O = optical trocar, X = additional trocars Image credit: Andy Wang

**Figure 6:**
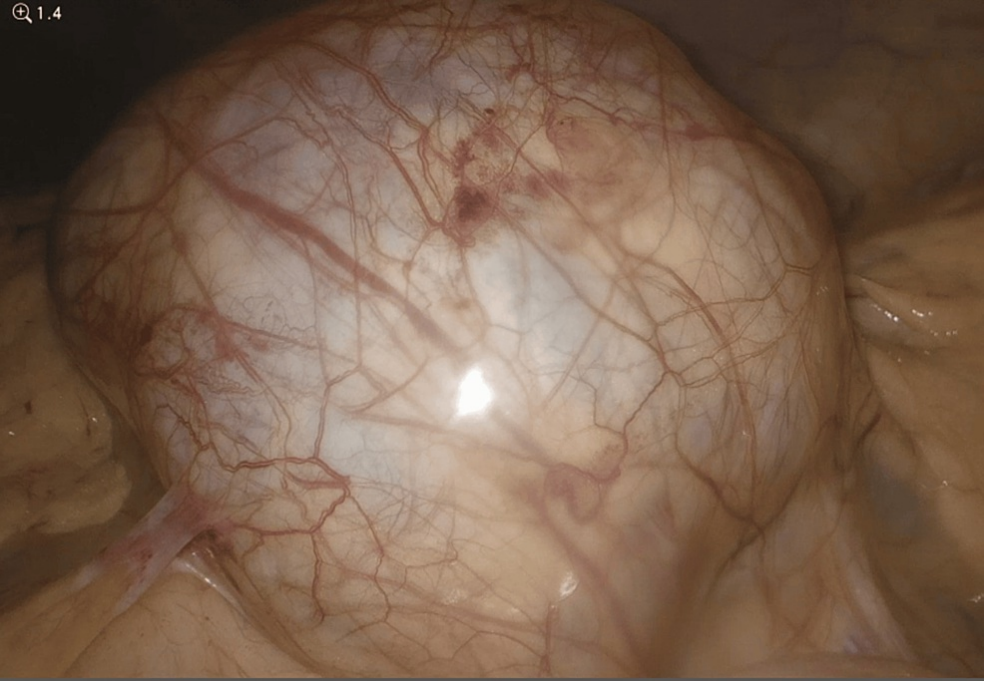
Initial laparoscopic view of para-aortic mass with overlying peritoneum without dissection

**Figure 7:**
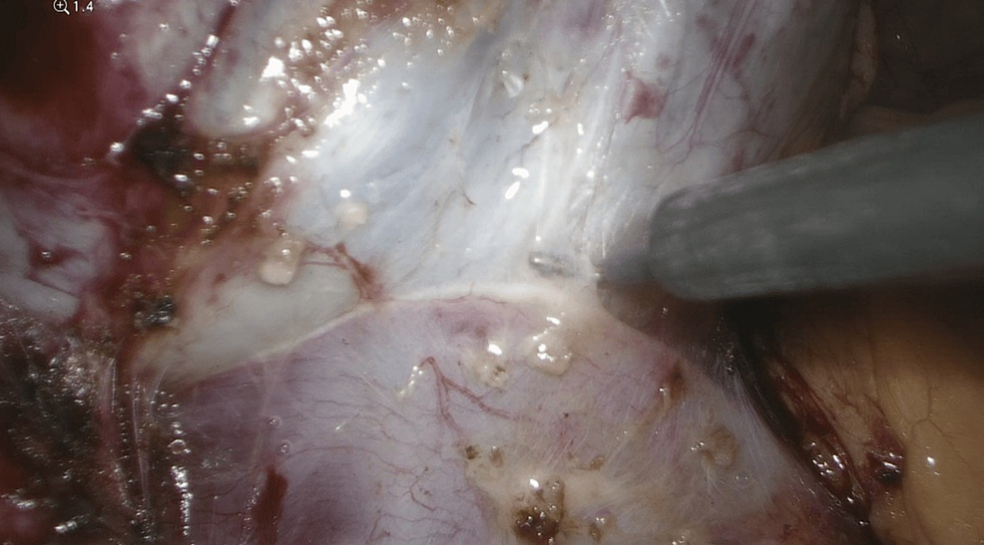
Separation of para-aortic mass from the common iliac vein using a monopolar cautery and blunt dissection

**Figure 8:**
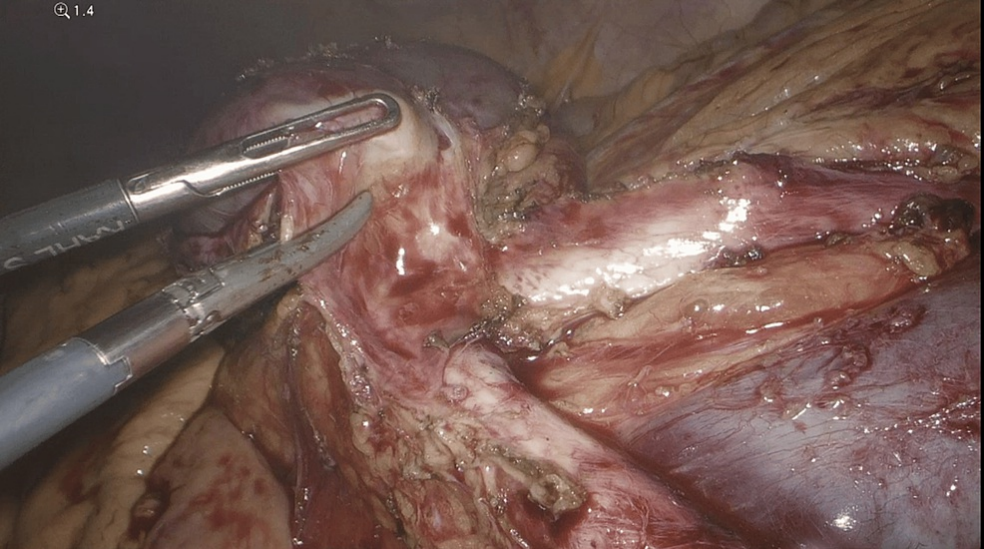
Dissection of the para-aortic mass off of the aortic bifurcation using bipolar cautery

Prior to surgery, the patient received a proper alpha blockade. No beta blockers were administered due to baseline bradycardia. In the operating room, the patient was correctly identified and positioned supine. Pneumoperitoneum was achieved with a Veress needle, and an optical 11-mm trocar was inserted in the right lower quadrant. After confirming no signs of injury from the Veress needle and the trocar, three additional 5-mm trocars were placed in the right upper, right middle, and suprapubic areas (Figure 5). The patient was then positioned in Trendelenburg. Upon entry into the abdomen, a large mass at the base of the aortic bifurcation was identified (Figure 6). Dissection began by making an incision adjacent to the mass to create a plane between it and its retroperitoneal attachments. This was performed using an ultrasonic device. The dense fiber capsule was then carefully dissected off the mass, and hemostasis was maintained by taking down numerous small veins in the capsule with an ultrasonic device. A meticulous dissection of the mass off the common iliac vein was performed using bipolar and monopolar cautery and blunt dissection (Figures 7, 8).

**Figure 9:**
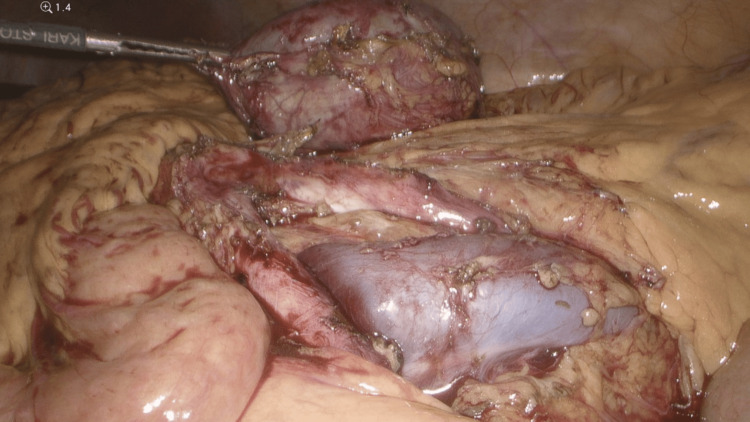
Completed dissection of the para-aortic mass off of the common iliac veins and aortic bifurcations with excellent hemostasis

The mass was separated from the aortic bifurcation by both blunt and sharp dissection, with no evidence of vascular complications (Figure 9). A 4-cm supraumbilical incision was made, and a small Alexis wound protector was placed. The specimen was successfully retrieved and pulled through the suprapubic incision. Following that, the abdominal cavity was inspected and noted to be hemostatic. All trocars were removed, and the laparoscopic incisions were closed with monofilament sutures and skin glue.

The patient tolerated the procedure well. He was monitored closely overnight. The following day, he was able to void spontaneously. He was discharged in good condition after one night of hospitalization. Two weeks after surgery, he was followed up and found to be healing appropriately. The pathology report of the resected specimen described the mass as a benign cyst, lined by benign mucinous epithelium and disorganized bundles of smooth muscle. Lymphoid aggregates were identified in the cyst wall. These findings were ultimately compatible with a tailgut cyst.

## Discussion

Tailgut cysts, also known as retrorectal or presacral cysts, are rare developmental anomalies derived from remnants of the embryonic hindgut [1]. While tailgut cysts are typically present in the retrorectal space, they can manifest at atypical locations, as evidenced by this case, posing diagnostic challenges [2-6]. Although tailgut cysts are generally benign, some grow progressively and lead to mass-related symptoms such as pelvic pain, constipation, or urinary dysfunction. Additionally, there is a risk of malignant transformation [7]. Therefore, it is important to urgently and accurately diagnose tailgut cysts. Despite advancements in diagnostic imaging, distinguishing tailgut cysts from other retrorectal lesions remains a challenge due to their similar radiological appearance. Clinical suspicion, combined with radiological findings and, in our case, histopathological examination, are crucial for an accurate diagnosis.

While the initial differential diagnoses of para-aortic lesions remain broad, including metastatic lymph nodes, dermoid cysts, teratomas, and tailgut cysts, this patient’s elevated serum metanephrine levels and hypertension alluded to a tumor of possible neuroendocrine origin. Therefore, we suspected a paraganglioma as the organ of Zuckerkandl. Unlike tailgut cysts, paragangliomas are neuroendocrine tumors derived from chromaffin cells in the adrenal medulla and autonomic ganglia. Macroscopically, they can present similarly as well-circumscribed masses with minimal invasion into vascular spaces if premalignant [8,9].

While our patient presented with clinical evidence and imaging suggestive of a neuroendocrine tumor, the final diagnosis was determined to be a tailgut cyst. In retrospect, although the patient’s serum metanephrines and catecholamines were elevated, his urine metanephrine was only mildly elevated. In fact, urine metanephrine has been demonstrated to be a superior diagnostic test with higher specificity for neuroendocrine tumors [10,11].

Ultimately, the diagnostic confirmation of the patient’s para-aortic mass was only possible after careful surgical exploration and a subsequent histopathological examination. Removal of these larger para-aortic masses is made difficult due to the proximity of the surrounding vasculature. Therefore, the surgical approach not only depends on the characteristics of the masses but also on patient-specific considerations and the experience of the surgeon [12].

The successful removal of such masses depends on multiple factors, including optimal trocar placement and meticulous dissection techniques, particularly given the intricate network of smaller vascular branches stemming from the aorta and proximity to the common iliac veins. In the context of our study, the trocar placement seen in Figure 5 allowed for optimal viewing of the surgical field. Typically, resecting a left lower quadrant mass requires a right upper quadrant optical trocar for triangulation [10]. However, due to the location of the para-aortic mass and the proximity of the common iliac veins located directly posterior and inferior to the mass, the surgeon determined a right lower quadrant optical trocar would provide greater visualization of the surrounding vasculature and therefore minimize vascular injury. After careful trocar placement, the surgeon should demonstrate proficiency in navigating the resection of this mass, employing a combination of monopolar and bipolar cautery alongside strategic blunt dissection. This multifaceted approach not only facilitated the thorough dissection of the mass but also ensured hemostasis throughout the procedure, as seen in Figure 9. By carefully managing the vascular branches of the aorta, one may effectively minimize the risk of intraoperative bleeding complications, underscoring the importance of surgical expertise and nuanced technique in achieving optimal outcomes in para-aortic mass resection.

In traditional surgical practice, the resection of larger (>5 cm) para-aortic masses has typically involved open surgery, a method associated with prolonged recovery times and increased risks of postoperative outcomes [11]. However, our case demonstrates the feasibility of a laparoscopic approach in managing larger para-aortic masses even when the tumor has adhered to major vascular structures when performed by an experienced surgeon. Our case highlights not only the utility of minimally invasive techniques in challenging anatomical cases but also emphasizes the advantages, including smaller incisions, less tissue trauma, reduced hospital stay, and overall improved patient outcomes [13,14]. Due to the skilled dissection and maintenance of hemostasis throughout the procedure, the patient required only one overnight hospital stay and follow-up in the clinic without any complications. A laparoscopic approach not only serves as a viable option but, in some cases, may become a preferred method for experienced surgeons.

## Conclusions

Tailgut cysts are rare developmental anomalies that can present a diagnostic challenge due to their diverse clinical manifestations and atypical locations. The differential diagnosis of para-aortic masses should include tailgut cysts, especially when encountering cystic lesions with heterogeneous components in imaging studies. Selecting a safe and effective surgical approach to remove para-aortic masses ultimately depends on various patient factors and the surgeon’s experience; however, we demonstrate that a laparoscopic approach is not only practical for smaller masses but also larger ones when performed by an experienced laparoscopic surgeon.
